# hsa_circ_0072309 Expression Profiling in Non-small-Cell Lung Carcinoma and Its Implications for Diagnosis and Prognosis

**DOI:** 10.3389/fsurg.2022.842292

**Published:** 2022-02-25

**Authors:** Yong Zhou, Zhongkai Tong, Xiaoxiao Zhu, Shaoqing Huang, Zhaoxing Dong, Zhenyue Ye

**Affiliations:** ^1^Respiratory Department, HwaMei Hospital, University of Chinese Academy of Sciences, Ningbo, China; ^2^Ningbo Institute of Life and Health Industry, University of Chinese Academy of Sciences, Ningbo, China

**Keywords:** hsa_circ_0072309, NSCLC, diagnosis, prognosis, GEO

## Abstract

Circular RNAs (circRNAs), which fall into the category of endogenous ncRNAs, are linked to disease progression of neoplastic diseases. Whereas, it remains uncharacterized regarding hsa_circ_0072309's function and implications in lung carcinoma (LC). Gene Expression Omnibus (GEO) database was utilized for identifying circRNAs with aberrantly expression in LC. qRT-PCR was responsible for determining hsa_circ_0072309 levels in lung adenocarcinoma (LAC). Also, its involvement in LC cell progression was investigated. Experimentally, hsa_circ_0072309 was identified as one of the most aberrantly down-regulated circRNAs in the GEO database (GSE101684 and GSE112214). qRT-PCR revealed notably down-regulated hsa_circ_0072309 in LAC tissue, which had a close association with adverse 3-year survival, as well as LNM and advanced TNM stage. Based on ROC, the AUC of hsa_circ_0072309 was determined to be 0.887, and its specificity and susceptibility can be improved by combined detection of either CYFRA21-1 or CEA. In a word, hsa_circ_0072309 is lowly expressed in lung cancer patients and the survival rate of lowly expressed patients is significantly lower, a candidate marker with prognostic utility for the disease.

## Introduction

Lung carcinoma (LC), as a prevalent respiratory malignancy (1). Epidemiological statistics reveal 4.3 million new cancer cases and an associated death toll of 2.9 million in 2018 in China, including 770,000 new LC cases and 690,000 associated deaths (2). Therefore, in the future, not only will the number of LC patients increase, but also the number of elderly patients (3). The predisposing factors of LC in the elderly include smoking, environmental exposure, ionizing radiation, and vocational diseases, heredity (4). However, due to the relatively concealed pathogenesis, the non-specific initial clinical manifestation, and the lack of good clinical diagnostic indicators, the disease has developed into the mid-late stage in some patients when they are hospitalized, resulting in losing the optimal time for operation (5). In addition, the long-term treatment cycle and dim outcomes of advanced LC aggravate the pressure of sufferers and the family economical load (6). Therefore, searching for a diagnostic index with high specificity and sensitivity is pressing to address this problem.

Recent evidence has indicated that non-coding RNAs (ncRNAs), closely linked to human life process, are vital in modulating a wide spectrum of illnesses, especially neoplastic diseases (7). As vital members of ncRNAs, miRNAs and lncRNAs have always been the focus of research (8). circRNAs, on the other hand, are recently found RNAs capable of forming loops that are covalently closed and continuous, with 3′ and 5′RNA ends binded, more favorable stability than linear types, and no polyadenosinic acid counterpart (9). hsa_circ_0072309, or LIFR, is located on chr5:38523520-38530768 (10), with its expression profiling being found in LC (11). But there is no research to prove its connection with patient prognosis and its diagnostic implications in non-small-cell lung carcinoma (NSCLC). Herein, we screened GSE101684 and GSE112214 microarrays and found that hsa_circ_0072309 had high levels in LC, suggesting its role of a candidate target for NSCLC.

Therefore, we have experimentally analyzed the expression of hsa_circ_0072309 in NSCLC patients and its diagnostic significance, providing a new potential target for clinical diagnosis and paving the way for our subsequent studies.

## Methods and Materials

### Microarray (GEO-Chip) Analysis

From GEO database (URL: https://www.ncbi.nlm.nih.gov/gds/), two microarrays (GSE101684 and GSE112214) were obtained. In the GSE101123 microarray, we found 4 tumor samples and 4 normal counterparts. And three tumor samples and three normal counterparts were found in the GSE112214 microarray. Then Series Matrix File(s) was downloaded, and GPL21825 and GPL19978 were sorted out into a matrix file. VLOOKUP function was utilized to correct the circRNA name and limma package to identify the circRNAs with differences (logFC = 1, *P* < 0.05). Volcano plots and heat maps were drawn.

### Clinical Data

Between February 2017 and February 2019, 46 NSCLC patients who received treatment in our hospital were included, with their peripheral blood samples collected before treatment. Meanwhile, peripheral blood specimens of 40 healthy people with concurrent physical examinations were used as control samples. Informed consents were got before patient enrollment. This study, in compliance with the Helsinki Declaration, has been ethically ratified by our hospital. The two arms were similar in age, sex and BMI (*P* > 0.05).

### Eligibility Criteria

Inclusion criteria: Diagnosis of NSCLC by imaging and pathology; In compliance with the TNM staging standard issued by AJCC (8th Edition) (12); Treatment-naive patients who had not received surgical resection, chemoradiotherapy, immunotherapy or molecular targeted therapy before participation.

Exclusion criteria: Other tumors; Estimated survival <3 months; No follow-up data; Incomplete case date.

### qRT-PCR Detection

Serum was analyzed in this study. TRIzol-extracted (Takara Bio, Shiga, Japan) total RNA from the collected serum was subjected to purity and concentration identification and the subsequent cDNA synthesis with use of the PrimeScript RT kit. Then the synthesized cDNA was subjected to amplification by PCR via the SYBR Premix Ex Taq kit, with the reaction system and reaction conditions configured following the instructions provided with the kit. The primers used are listed in Table 1. hsa_circ_0072309 expression normalized with β-actin (endogenous control) was obtained via the formula 2^−ΔΔCT^[ΔCT(test) = CT(target, test)–CT(ref, test)ΔCT(calibrator) = CT(target, calibrator)–CT(ref, calibrator), ΔΔCT = ΔCT(test)–ΔCT(calibrator)]. In this experiment, the ABI 7500 PCR system was used for analysis.

**Table 1:** The top five up- down-regulated circRNAs in the GSE101684 microarray.

**Symbol**	**logFC**	**AveExpr**	***t*-value**	***P*-Value**	**adj.P.Val**	**β**
**Up**						
hsa_circ_0072088	2.830	10.003	5.380	4.212E-04	0.011	0.339
hsa_circ_00723098	2.828	11.026	8.856	8.780E-06	0.003	4.135
hsa_circ_0067301	2.689	9.446	7.046	5.560E-05	0.005	2.358
hsa_circ_0001806	2.571	13.307	7.611	3.010E-05	0.004	2.955
hsa_circ_0006349	2.459	12.740	9.572	4.590E-06	0.003	4.739
**Down**						
hsa_circ_0000317	−2.627	10.286	−5.631	3.033E-04	0.009	0.670
hsa_circ_0001640	−2.057	7.020	−4.820	9.056E-04	0.016	−0.433
hsa_circ_0013480	−2.042	8.534	−6.318	1.290E-04	0.006	1.525
hsa_circ_0000320	−2.005	9.213	−4.839	8.815E-04	0.016	−0.406
hsa_circ_0000319	−1.938	7.523	−5.343	4.430E-04	0.012	0.289

### CEA and CYFRA21-1 Detection

For all experiments, blood samples were collected from an elbow vein in the early morning on an empty stomach prior to the start of treatment. Serum tumor markers: 10 ml of blood was collected. CEA and CYFRA21-1 were measured using a Beckman Coulter AU5800 automated biochemistry analyser.

### Endpoints

Primary endpoints: The differential circRNAs in GSE101684 and GSE112214 microarrays were analyzed and heat maps and volcano plots were drawn. Serum and carcinoma tissue hsa_circ_0072309 expression in cases was tested. ROC curves were drawn to visualize hsa_circ_0072309's diagnostic implications in NSCLC.

Secondary endpoints: The connection between hsa_circ_0072309 and patient clinical data was analyzed. In addition, patients were followed up for 3 years to observe the connection between hsa_circ_0072309 and patient prognosis.

### Statistical Processing

The above index data were input into SPSS21.0 and GraphPad Prism 8.0 for statistical processing and plotting, respectively. Quantitative variables, described in (Mean ± SD), were analyzed via the independent samples *T* test. While represented by n, categorical variables were examined using the χ^2^ test. ROC curves were used for assessing the diagnostic implications of serum hsa_circ_0072309. Patient survival was evaluated by the K-M algorithm. The R software limma package was used to analyse the differential genes, the library package to map the differential gene volcanoes and the heatmap package to map the differential gene heat map. Significance was set at the probability (*P*) level < 0.05.

## Results

### GEO-Chip Analysis

**Figure 1:**
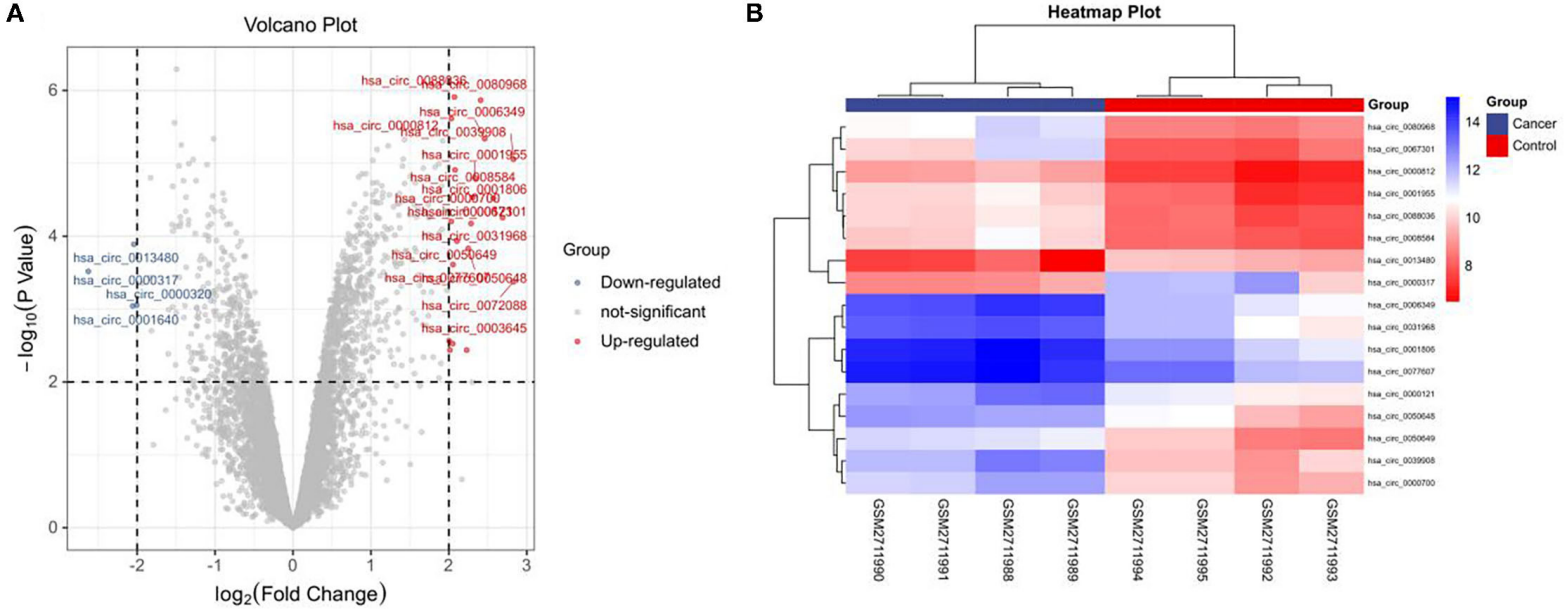
Volcanic plot and heat map of circRNAs with aberrant expression in the GSE101684 microarray. **(A)**. Volcanic plot of circRNAs with aberrant expression in the GSE101684 microarray: Red, blue and gray represent those with high, low, and undifferential expression, respectively. **(B)**. Heat map of circRNAs with aberrant expression in the GSE101684 microarray.

**Figure 2:**
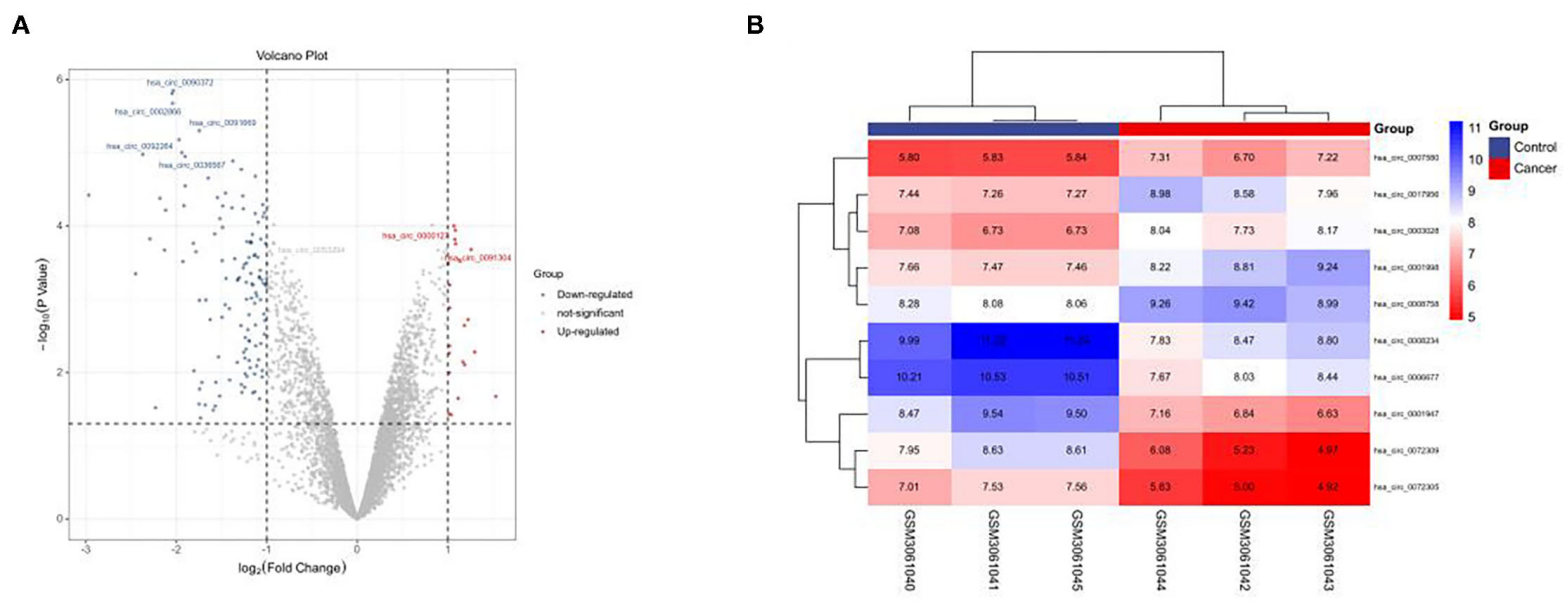
Volcanic plot and heat map of circRNAs with aberrant expression in the GSE112214 microarray. **(A)**. Volcanic plot of differentially expressed circRNAs in the GSE112214 microarray: Red, blue and gray indicate those with high, low, and undifferential expression, respectively. **(B)**. Heat map of circRNAs with aberrant expression in the GSE112214 microarray.

**Figure 3:**
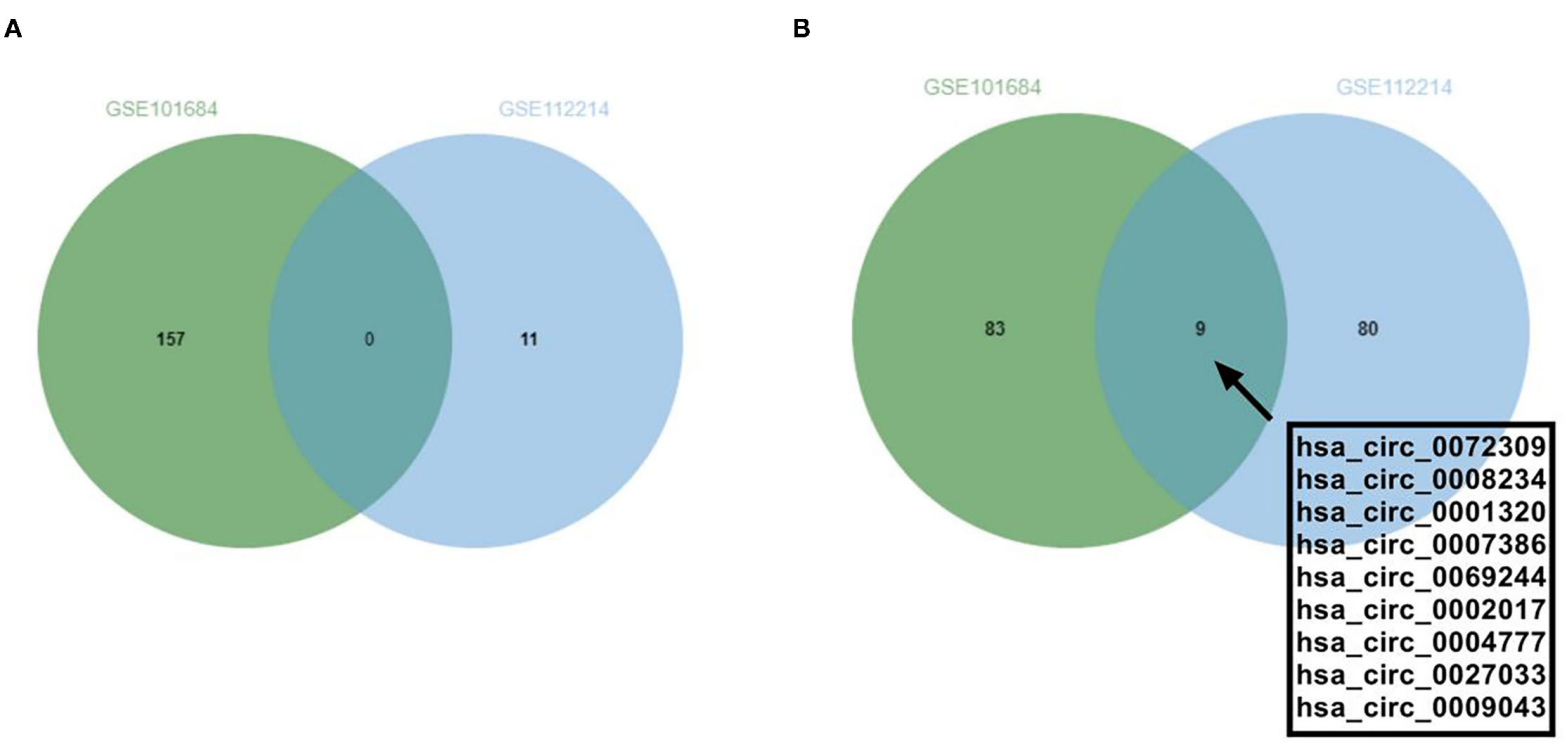
Shared differentially expressed circRNAs in GSE101684 and GSE112214 microarrays. **(A)**. The shared highly expressed circRNAs in GSE101684 and GSE112214 microarrays. **(B)**. The shared low-expressed expressed circRNAs in GSE101684 and GSE112214 microarrays.

In this study, the GSE101684 microarray was analyzed, and 249 differential circRNAs were identified, of which 157 were over-expressed and 92 were under-expressed (Table 1, Figures 1A,B). One hundred differential circRNAs were found through analyzing the GSE112214 microarray, including 11 over-expressed and 89 were under-expressed circRNAs (Table 2, Figures 2A,B). In order to find shared differentially expressed circRNAs, we drew a Venn diagram to find zero shared highly expressed circRNAs in the two microarrays while nine common lowly expressed circRNAs (Figures 3A,B). hsa_circ_0072309 with the most significant difference and the largest LogFC was selected for follow-up study.

**Table 2:** The top five up- down-regulated circRNAs in the GSE112214 microarray.

**Symbol**	**logFC**	**AveExpr**	***t*-value**	***P*-value**	**adj.P.Val**	**β**
**Up**						
hsa_circ_0007580	1.257	6.451	7.041	2.083E-04	1.769E-02	1.232
hsa_circ_0001998	1.223	8.145	4.843	1.894E-03	3.874E-02	−1.045
hsa_circ_0017956	1.183	7.915	4.684	2.276E-03	4.295E-02	−1.237
hsa_circ_0003028	1.132	7.414	6.669	2.911E-04	1.974E-02	0.892
hsa_circ_0008758	1.087	8.681	7.245	1.743E-04	1.568E-02	1.412
**Down**						
hsa_circ_0072309	−2.968	6.912	−9.212	3.770E-05	1.020E-02	2.918
hsa_circ_0008234	−2.450	9.592	−6.210	4.486E-04	2.251E-02	0.450
hsa_circ_0006677	−2.372	9.231	−11.186	1.050E-05	6.183E-03	4.104
hsa_circ_0001947	−2.293	8.022	−7.425	1.495E-04	1.568E-02	1.566
hsa_circ_0072305	−2.181	6.274	−9.070	4.170E-05	1.020E-02	2.822

### hsa_circ_0072309 in Cases

**Figure 4:**
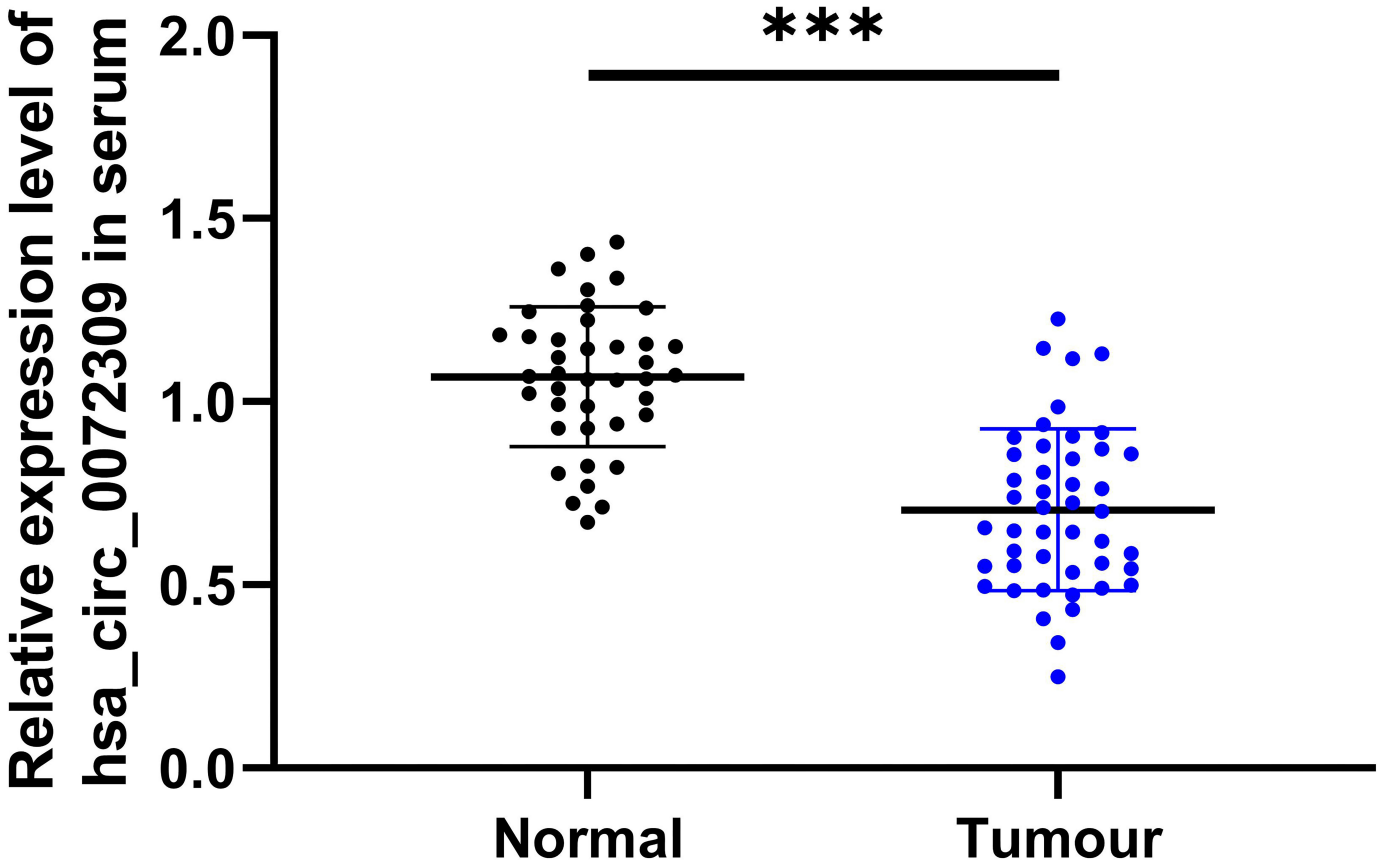
Serum hsa_circ_0072309 in LC patients. ***P < 0.001.

We compared serum hsa_circ_0072309 levels between cases and controls and found statistically higher hsa_circ_0072309 levels in cases (Figure 4, *P* < 0.001).

### Connection Between hsa_circ_0072309 and Patient Clinical Data

Based on median hsa_circ_0072309 expression, cases were subgrouped (high/expression groups, 23 cases each). The comparison of clinical data revealed an evidently elevated chance of developing III-IV LC and lymph node metastasis (LNM) in patients with under-expressed hsa_circ_0072309 (Table 3, *P* < 0.05).

**Table 3:** Connection between hsa_circ_0072309 and patient clinical data.

**Variables**		**Relative expression of hsa_circ_0072309**		***P*-value**
		**High expression (*n* = 23)**	**Low expression (*n* = 23)**	
Age				0.552
	≥60 (*n* = 20)	9	11	
	<60 (*n* = 26)	14	12	
Gender				0.555
	Male (*n* = 22)	12	10	
	Female (*n* = 24)	11	13	
Tumor size (cm)				0.546
	≥3 (*n* = 18)	10	8	
	<3 (*n* = 28)	13	15	
Clinical staging				0.003
	I-II (*n* = 26)	18	8	
	III-IV (*n* = 20)	5	15	
LNM				0.013
	With (*n* = 16)	4	12	
	Without (*n* = 30)	19	11	
Tumor types				0.767
	Squamous cell carcinoma (*n* = 21)	11	10	
	Adenocarcinoma (*n* = 25)	12	13	

### Diagnostic Implications of hsa_circ_0072309 in NSCLC Patients

**Figure 5:**
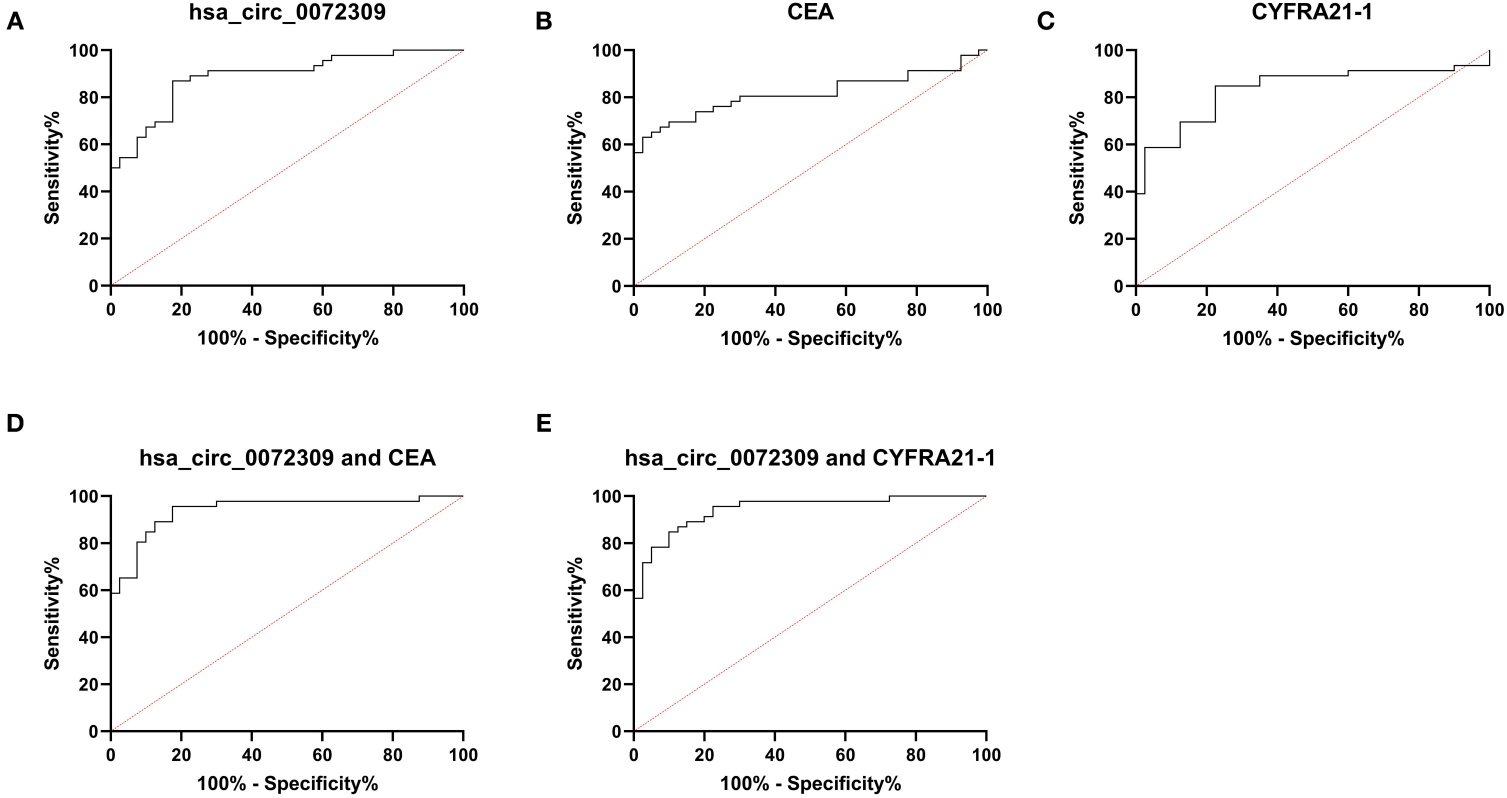
hsa_circ_0072309 for NSCLC diagnosis by ROC analysis. **(A)**. hsa_circ_0072309's implications for NSCLC diagnosis by ROC. **(B)**. CEA's implications for NSCLC diagnosis by ROC. **(C)**. CYFRA21-1's implications for NSCLC diagnosis by ROC. **(D)**. Diagnosistic implications of hsa_circ_0072309+CEA for NSCLC by ROC. **(E)**. Diagnosistic implications of hsa_circ_0072309+CYFRA21-1 for NSCLC by ROC.

ROC curves were drawn to understand hsa_circ_0072309's diagnostic implications in NSCLC patients. Through analysis, hsa_circ_0072309's AUC, specificity and susceptibility were determined to be 0.887, 82.50, and 86.95%, respectively, which were evidently higher than those of CEA (0.815, 97.50, and 63.04%) and CYFRA21-1 (0.834, 77.50, and 84.78%). Further, hsa_circ_0072309 could improve the sensitivity and specificity of CEA and CYFRA21-1 through joint prediction (Table 4, Figure 5).

**Table 4:** ROC curve parameters.

**Variables**	**AUC**	**95 CI%**	**Specificity**	**Sensitivity**	**Youden index**	**Cut-off**
hsa_circ_0072309	0.887	0.818–0.956	82.50%	86.95%	69.45%	0.921
CEA	0.815	0.720–0.911	97.50%	63.04%	60.54%	6.001
CYFRA21-1	0.834	0.743–0.924	77.50%	84.78%	62.28%	3.128
hsa_circ_0072309+CEA	0.940	0.889–0.990	82.50%	95.65%	78.15%	0.337
hsa_circ_0072309+CYFRA21-1	0.944	0.898–0.989	90.00%	84.78%	74.78%	0.614

### Cox Regression Analysis

**Figure 6:**
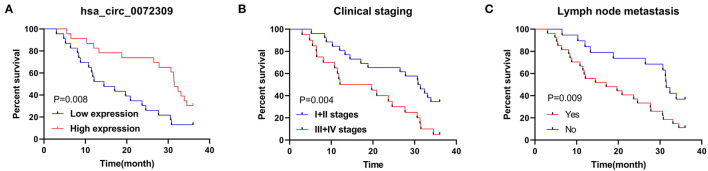
K-M survival curve of factors with differences in univariate analysis. **(A)**. Three-year survival of NSCLC patients with up- and down-regulated hsa_circ_0072309 by K-M curves. **(B)**. Three-year survival of NSCLC patients with I-II or III-IV LC by K-M curves. **(C)**. Three-year survival of NSCLC patients with and without lymph node metastasis by K-M curves.

Finally, we conducted statistics on the 3-year survival of patients and Cox regression analysis by combining patient's medical records. Univariate analysis revealed that clinical stage, LNM, hsa_circ_0072309 were prognostic factors for NSCLS patients (Figure 6). Furthermore, multivariate Cox regression analysis by further incorporating differentially expressed factors in univariate analysis identified the independence of LNM and hsa_circ_0072309 as prognostic factors for NSCLS (Table 5).

**Table 5:** Cox regression.

**Factors**	**Univariate**			**Multivariate**		
	***P*-value**	**HR**	**95%CI**	***P*-value**	**HR**	**95%CI**
Age	0.708	0.881	0.455–1.707			
Gender	0.749	0.898	0.466–1.731			
Tumor size	0.268	1.482	0.739–2.975			
Clinical staging	0.005	2.582	1.323–5.042	0.180	1.666	0.790–3.515
Lymph node metastasis	0.004	0.364	0.185-0.719	0.010	0.399	0.198–0.804
Tumor types	0.249	1.479	0.760–2.876			
hsa_circ_0072309	0.010	2.426	1.234–4.767	0.025	2.219	1.104–4.460

## Discussion

Due to the current lack of basic biological understanding of NSCLC, the feasible biomarkers for detection and effective medicines for treatment are lacking (13, 14). Relevant literature has revealed a close connection between the survival of LC patients and staging, with the 5-year survival rate decreasing from 82 to 6% as the staging (IA-IV) increases (15). However, early diagnosis is too hard given the non-specific early symptoms (16). Consequently, most cases are confirmed at the late stage when the best treatment opportunity has been missed (17). Therefore, finding effective diagnostic markers is beneficial to improve patient prognosis and survival, which is also a current clinical challenge.

CircRNA are recently found endogenous ncRNAs with closed-loop structure (18). Because of their high tissue specificity and stablility in structure, they are less prone to digestion by exonuclease (RNase) (19), which also results in circRNAs being potentially favorable biomarkers for diagnosing and predicting outcomes of various illnesses (20). Earlier, research has revealed the close association between circRNA-100876 and prognosis of colorectal cancer (21). Another research identified markedly upregulated hsa_circ_0004585 in colorectal cancer and its function as a feasible biomarker for the disease (22). All these studies suggest that circRNAs are feasible biomarkers for cancer diagnosis.

For the purpose of determining the expression of circRNAs in NSCLC, GSE101684 and GSE112214 microarray datasets were retrieved through the GEO database in this study for analysis. Additionally, qRT-PCR was carried out for identifying the circRNAs with aberrant expression in clinical samples. It identified underexpressed hsa_circ_0072309 in cases, which agreed the findings of Mo et al. (11). However, their study did not further analyze hsa_circ_0072309's clinical application in NSCLC. Through analysis, we further confirmed a notably increased probability of LNM and clinical stage III-IV in patients with hsa_circ_0072309 underexpression. Therefore, it may interfere with NSCLC cell growth, progression and migration. Furthermore, we probed into hsa_circ_0072309's clinical implications in diagnosing NSCLC. As indicated by ROC curve analysis, hsa_circ_0072309's AUC was 0.887, notably higher than that of CEA and CYFRA21-1. AUC is considered to be the best index to evaluate the diagnostic value, with a value of <0.5, 0.5- <0.7, 0.7- <0.9 and ≥0.9 indicating no diagnostic value, lower accuracy, certain accuracy, and higher accuracy respectively. This suggests that hsa_circ_0072309 is a feasible diagnostic index for NSCLC (23). We then performed joint prediction based on Logistic regression, and found improved sensitivity of CEA and CYFRA21-1 by hsa_circ_0072309. Finally, we discussed the connection between hsa_circ_0072309 and patient prognosis. As shown by Cox regression analysis, hsa_circ_0072309 was independently linked to patient prognosis, suggesting its potential as an essential marker for adverse prognosis in NSCLC patients.

This research experimentally confirmed down-regulated_circ_0072309 in NSCLC and its potential to be a marker with diagnosis and prognosis utility for NSCLC. However, there are some limitations to be addressed. We only collected samples from LC patients, but not patients with benign lung diseases. The occurrence of LC is a long process, whether hsa_circ_0072309 can distinguish benign lung diseases from LC remains to be defined. Second, this study has a single sample type. Recent studies have found that circRNAs exist in exosomes, peripheral blood mononuclear cells and alveolar fluid. Whether hsa_circ_0072309 is more significantly differentially expressed in other samples needs further verification. Third, this time, the mechanism of hsa_circ_0072309 has not been deeply explored, and it is hoped that the later expansion through bioinformatics analysis will lay the foundation for our follow-up basic experiments.

In conclusion, hsa_CIRC_0072309 is a candidate marker with prognostic utility in lung cancer patients with low expression and a significantly lower survival rate in patients with low expression.

## Data Availability Statement

The datasets presented in this study can be found in online repositories. The names of the repository/repositories and accession number(s) can be found in the article/supplementary material.

## Author Contributions

YZ and ZT are the mainly responsible for the writing of the article. XZ is mainly responsible for research design. SH is mainly responsible for data analysis. ZD and ZY are responsible for the guidance of the entire research. All authors contributed to the article and approved the submitted version.

## Funding

This work was supported by Natural Science Foundation of Zhejiang Province (No. LQ21H160014), Medical Health Science and Technology Project of Zhejiang Provincial Health Commission (No. 2018KY689), and Ningbo Health Branding Subject Fund (No. PPXK201805).

## Conflict of Interest

The authors declare that the research was conducted in the absence of any commercial or financial relationships that could be construed as a potential conflict of interest.

## Publisher's Note

All claims expressed in this article are solely those of the authors and do not necessarily represent those of their affiliated organizations, or those of the publisher, the editors and the reviewers. Any product that may be evaluated in this article, or claim that may be made by its manufacturer, is not guaranteed or endorsed by the publisher.

## References

[1] Nasim F Sabath BF Eapen GA. Lung cancer. Med Clin North Am. (2019) 103:463–73. 10.1016/j.mcna.2018.12.006 30955514

[2] Bray F Ferlay J Soerjomataram I Siegel RL Torre LA Jemal A. Global cancer statistics 2018: GLOBOCAN estimates of incidence and mortality worldwide for 36 cancers in 185 countries. CA Cancer J Clin. (2018) 68:394–424. 10.3322/caac.21492 30207593

[3] Schabath MB Cote ML. Cancer progress and priorities: lung cancer. Cancer Epidemiol Biomarkers Prev. (2019) 28:1563–79. 10.1158/1055-9965.EPI-19-0221 31575553 PMC6777859

[4] Jones GS Baldwin DR. Recent advances in the management of lung cancer. Clin Med. (2018) 18:s41–6. 10.7861/clinmedicine.18-2-s41 29700092 PMC6334032

[5] Hoy H Lynch T Beck M. Surgical treatment of lung cancer. Crit Care Nurs Clin North Am. (2019) 31:303–13. 10.1016/j.cnc.2019.05.002 31351552

[6] Ruiz-Cordero R Devine WP. Targeted therapy and checkpoint immunotherapy in lung cancer. Surg Pathol Clin. (2020) 13:17–33. 10.1016/j.path.2019.11.002 32005431

[7] Zhu J Zhang X Gao W Hu H Wang X Hao D. lncRNA/circRNAmiRNAmRNA ceRNA network in lumbar intervertebral disc degeneration. Mol Med Rep. (2019) 20:3160–74. 10.3892/mmr.2019.10569 31432173 PMC6755180

[8] Bushati N Cohen SM. microRNA functions. Annu Rev Cell Dev Biol. (2007) 23:175–205. 10.1146/annurev.cellbio.23.090506.123406 17506695

[9] Patop IL Kadener S. circRNAs in cancer. Curr Opin Genet Dev. (2018) 48:121–7. 10.1016/j.gde.2017.11.007 29245064 PMC5877416

[10] Yan L Zheng M Wang H. Circular RNA hsa_circ_0072309 inhibits proliferation and invasion of breast cancer cells via targeting miR-492. Cancer Manag Res. (2019) 11:1033–41. 10.2147/CMAR.S186857 30774431 PMC6349082

[11] Mo WL Deng LJ Cheng Y Yu WJ Yang YH Gu WD. Circular RNA hsa_circ_0072309 promotes tumorigenesis and invasion by regulating the miR-607/FTO axis in non-small cell lung carcinoma. Aging. (2021) 13:11629–45. 10.18632/aging.202856 33879631 PMC8109101

[12] Koul R Rathod S Dubey A Bashir B Chowdhury A. Comparison of 7th and 8th editions of the UICC/AJCC TNM staging for non-small cell lung cancer in a non-metastatic North American cohort undergoing primary radiation treatment. Lung Cancer. (2018) 123:116–20. 10.1016/j.lungcan.2018.06.029 30089581

[13] Skrickova J Kadlec B Venclicek O Merta Z. Lung cancer. Cas Lek Cesk. (2018) 157:226–36. 30441934

[14] Duruisseaux M Esteller M. Lung cancer epigenetics: From knowledge to applications. Semin Cancer Biol. (2018) 51:116–28. 10.1016/j.semcancer.2017.09.005 28919484

[15] Milano AF. 20-Year comparative survival and mortality of cancer of the stomach by age, sex, race, stage, grade, cohort entry time-period, disease duration & selected ICD-O-3 oncologic phenotypes: a systematic review of 157,258 cases for diagnosis years 1973-2014: (SEER^*^Stat 8.3.4). J Insur Med. (2019) 48:5–23. 10.17849/insm-48-1-1-19.1 31609640

[16] Alexander M Kim SY Cheng H. Update 2020: management of non-small cell lung cancer. Lung. (2020) 198:897–907. 10.1007/s00408-020-00407-5 33175991 PMC7656891

[17] Oudkerk M Liu S Heuvelmans MA Walter JE Field JK. Lung cancer LDCT screening and mortality reduction - evidence, pitfalls and future perspectives. Nat Rev Clin Oncol. (2021) 18:135–51. 10.1038/s41571-020-00432-6 33046839

[18] Shi Y Jia X Xu J. The new function of circRNA: translation. Clin Transl Oncol. (2020) 22:2162–9. 10.1007/s12094-020-02371-1 32449127

[19] Li S Teng S Xu J Su G Zhang Y Zhao J. Microarray is an efficient tool for circRNA profiling. Brief Bioinform. (2019) 20:1420–33. 10.1093/bib/bby006 29415187

[20] Liu J Xue N Guo Y Niu K Gao L Zhang S. CircRNA_100367 regulated the radiation sensitivity of esophageal squamous cell carcinomas through miR-217/Wnt3 pathway. Aging. (2019) 11:12412–27. 10.18632/aging.102580 31851619 PMC6949088

[21] Zhou GR Huang DP Sun ZF Zhang XF. Characteristics and prognostic significance of circRNA-100876 in patients with colorectal cancer. Eur Rev Med Pharmacol Sci. (2020) 24:11587–93. 10.26355/eurrev_202011_23801 33275225

[22] Tian J Xi X Wang J Yu J Huang Q Ma R. CircRNA hsa_circ_0004585 as a potential biomarker for colorectal cancer. Cancer Manag Res. (2019) 11:5413–23. 10.2147/CMAR.S199436 31354349 PMC6573007

[23] Kim J Hwang IC. Drawing guidelines for receiver operating characteristic curve in preparation of manuscripts. J Korean Med Sci. (2020) 35:e171. 10.3346/jkms.2020.35.e171 32567255 PMC7308134

